# Evaluation of Heartwood Extracts Combined with Linseed Oil as Wood Preservatives in Field Tests in Southern Mississippi, USA

**DOI:** 10.3390/insects12090803

**Published:** 2021-09-08

**Authors:** Babar Hassan, Mark E. Mankowski, Grant T. Kirker

**Affiliations:** 1 National Center for Timber Durability and Design Life, University of Sunshine Coast, Brisbane, QLD 4102, Australia; 2 USDA-FS, Forest Products Laboratory, 201 Lincoln Green, Starkville, MS 39759, USA; mark.e.mankowski@usda.gov; 3 USDA-FS, Forest Products Laboratory, 1 Gifford Pinchot Dr, Madison, WI 53726, USA; gkirker@fs.fed.us

**Keywords:** plant oils, wood extracts, wood preservation, natural compounds, biodeterioration, field tests, AWPA E7, AWPA E26, *Reticulitermes*, decay fungi

## Abstract

**Simple Summary:**

Wood is a sustainable building material with a wide range of applications. Being of biological origin, wood is subject to degradation by several organisms, including termites and decay fungi. Chemical preservatives are often used to protect the wood from biodegradation but concerns regarding the overall safety and availability of chemical preservatives has stimulated research into alternative treatment methods. One approach is transferring heartwood extracts of durable wood species to non-durable wood due to their intrinsic antimicrobial properties, renewability, and perceived lower impact on the environment. Although they are not free of limitations, their efficacy can potentially be improved with methods such as co-impregnating wood with other plant-based hydrophobic chemicals. In the current study, we used heartwood extracts of four wood species and linseed oil to impregnate two non-durable wood species. Results show that co-impregnation with individual heartwood species extract, and oil increased the resistance of non-durable wood against termites and decay fungi. However, their effectiveness to protect was less than chemical preservatives. Further research is needed to examine single-extract component isolates combined with oils as well as possible synergists or co-biocides to enhance overall performance.

**Abstract:**

Heartwood extracts of naturally durable wood species are often evaluated as alternatives to chemical wood preservatives, but field data from long-term performance testing are lacking. The current study evaluated the long-term (five-year) performance of two non-durable wood species treated with heartwood extracts of either *Tectona grandis*, *Dalbergia sissoo*, *Cedrus deodara*, or *Pinus roxburghii* alone or combined with linseed oil. Stakes (45.7 × 1.9 × 1.9 cm) and blocks (12.5 × 3.75 × 2.5 cm) cut from the sapwood of cottonwood and southern pine were vacuum-pressure impregnated with the individual heartwood species extract, linseed oil, or a mixture of each individual wood extract and linseed oil. For comparison, solid heartwood stakes and blocks of the wood species used to obtain extracts were also included in the tests. All samples were exposed for five years to decay and termites at a test site in southern Mississippi using ground contact (AWPA E7) and ground proximity (AWPA E26) tests. Results showed that extract-oil mixtures imparted higher termite and decay resistance in cottonwood and southern pine than linseed oil only or the individual heartwood species extract in both tests. However, these treatments were as not effective as to commercially used wood preservatives, copper naphthenate (CuN) or disodium octaborate tetrahydrate (DOT) in either test. Moreover, solid heartwood *P. roxburghii* stakes were completely decayed and attacked by termites after five years in the ground contact test. In contrast, *C. deodara* stakes were slightly attacked by termites and moderately attacked by decay fungi. However, *T. grandis* and *D. sissoo* stakes showed slight to superficial attack by termites and decay fungi in ground contact test. In contrast, *T. grandis* and *D. sissoo* blocks showed slight decay fungi attack in above-ground tests. However, termites did not attack *T. grandis*, *D. sissoo*, and *C. deodara* blocks. However, decay fungi moderately attacked *C. deodara* blocks, and *P. roxburghii* blocks were severely attacked by decay fungi and termites in the above-ground test.

## 1. Introduction

Wood is one of the most used versatile and sustainable construction materials globally. However, it is subject to biodeterioration by decay fungi and insects, and this susceptibility requires appropriate protection to extend its service life. Traditional chemical wood preservatives have been used to control the bio-susceptibility of wood for decades due to their low cost and proven efficacy in varying environmental habitats. In some countries, increased regulatory pressures have increased attention toward the development of more environmentally sustainable wood preservative systems. Preservative treated wood contains components that may be toxic to non-target organisms if released into the environment in sufficient quantities. Therefore, wood treated with some chemical preservatives is categorized as hazardous waste in some countries [1,2]. These considerations have created demand for the development of alternative methods that are eco-friendly and long lasting. As an alternative, natural preservatives (preservatives derived from natural products, particularly botanicals) have gained increasing attention due to their intrinsic antimicrobial properties, renewability, and perceived lower impact on the environment [3].

The heartwood of certain wood species is resistant to biodeterioration due to the sequestration of extracts as a tree matures and ages. These extracts are non-structural components of the wood that constitute a small fraction of wood microstructure [3]. Previous studies showed that heartwood extracts of many species have strong fungicidal, insecticidal, repellent, antifeedant, and antioxidant properties [4,5,6,7,8]. Transfer of heartwood extracts from durable species to non-durable wood is a promising approach for developing new wood protectant systems. Previous studies show that pressure treating non-durable wood with heartwood extracts can protect non-durable wood species against fungi and termite attack [9,10,11,12,13]. However, heartwood extracts′ potential to be as effective as synthetic chemicals in protecting wood is questionable. Lower retention of extracts in impregnated wood, susceptibility to biodegradation, and high cost are the primary concerns [3].

Combining other natural compounds such as hydrophobic plant oils with heartwood extracts can be one method to increase their efficacy. Like wood extracts, oils obtained from seeds and foliage of many plant species exhibit insecticidal, antimicrobial, antioxidant, antifeedant, and repellent properties [3]. Several plant oils have been used to impregnate non-durable wood for protection against decay fungi and termites [14,15,16,17,18]. Examples are neem [19], linseed [18], rapeseed [20], and tall oil [21]. Linseed oil has long been used as a wood preservative. It is a very effective wood protectant when combined with other organic biocides [3]. Plant oils are water repellent and can retain other biocides to help transfer them deep into wood during impregnation and, thus, increase the efficiency of the wood preservative being used [18,20].

The combination of two natural biocides can increase their efficacy. Synergy with an additive formulation can reduce production costs and increase effectiveness against wood-degrading organisms [10]. In the current study, we combined biocidal heartwood extracts and linseed oil to impregnate two non-durable wood species to develop a multi-component, naturally derived wood preservative system and tested it in the field for five years.

## 2. Materials and Methods

### 2.1. Wood Material and Preparation of Extracts

The heartwood of *Tectona grandis* L.f., *Dalbergia sissoo* Roxb. ex DC., *Cedrus deodara* (Roxb.) G. Don, and *Pinus roxburghii* Sarg. was selected for the current studies. Selected wood species are commonly used in Pakistan as naturally durable species, and their extract′s preservative potential has not been tested apart from *T. grandis*. Further, properties of the selected species are given in Hassan et al. [10]. The heartwood of marine grade *T. grandis* was acquired from a supplier in the United States (McIlvain, Pittsburgh, PA, USA). While heartwood of *D. sissoo*, *C. deodara*, and *P. roxburghii* was sourced from a timber market in Faisalabad, Pakistan, and shipped to Starkville, MS. Heartwood of all species was cut into boards (457 × 127 × 19 mm) and air dried for four weeks. For the preparation of extracts, we followed Hassan et al. [7]. Wood shavings were air-dried in the laboratory for four weeks and placed in 12–15 g batches in each of 20 Soxhlet extractors and processed according to ASTM D1105-96 (Standard Test Method for Preparation of Extractive-Free Wood) using 300 mL of ethanol:toluene (2:1) as the solvent system [22]. Further details about preparation and storage of extracts are given in Hassan et al. [9]. Obtained extracts were further diluted using mixture of ethanol/toluene (2:1) to prepare final concentrations for the treatment of non-durable wood.

Boiled linseed (*Linum usitatissimum* L.) oil was purchased Sunnyside Corporation, Wheeling, IL which was further diluted up to 20% concentration using ethanol/toluene (2:1) prior to treatment of non-durable wood

### 2.2. Non-Durable Wood Species and Treatment Process

Cottonwood and southern pine sapwood were selected as non-durable wood species to treat with extracts and linseed oil. Boards of both species were acquired locally (Madison, WI, USA) and cut into stakes (parallel to grain) measuring 45.7 × 1.9 × 1.9 cm to initiate the AWPA E7 ground contact field stake test. Sapwood of both species was also cut into blocks measuring 12.5 × 3.75 × 2.5 cm for a covered ground proximity test following AWPA E26. Blocks and stakes of both species were treated using vacuum pressure by following methods described by Hassan et al. [10]. Stakes and blocks of non-durable wood species were conditioned at 33 °C and 62 ± 3% RH prior to treatment. All blocks and stakes were treated with 7.5 mg mL^−1^ extract of each durable wood at 7.5 mg mL^−1^ concentration, a mixture of extract and linseed oil in which extract concentration was 4.25 mg mL^−1^, while oil concentration was 20%, and linseed oil at 20% concentration alone. Blocks and stakes of nondurable wood species were treated with these treatment solutions separately in a vacuum pressure chamber. Blocks and stakes of both non-durable wood species were treated with ethanol/toluene only as a control treatment. Full cell vacuum pressure with an initial vacuum at 91.43 kPa gauge for 30 min, and then the pressure at 1034-kPa gauge was applied for 60 min to treat all test samples. For positive controls in ground contact tests (AWPA E7), stakes of both non-durable wood species were treated with 9.6% oil-based copper naphthenate (CuN) which is commercially used for ground contact applications against termites and decay fungi. Blocks of both non-durable wood species for the ground proximity tests (AWPA E26) were treated with a 67% borate solution (disodium octaborate tetrahydrate (DOT); as Tim-Bor) for the positive chemical control [23]. Retentions of cottonwood and southern pine impregnated with linseed oil (20%), heartwood extracts (7.5 mg mL^−1^), or a mixture of each heartwood extract and oil separately (4.25 mg mL^−1^ + 20% oil) are given in Table 1.

Additionally, heartwood stakes and blocks of the four durable test species were also exposed in the field for comparison.

### 2.3. Field Testing

Conditioned (33 °C, 62 ± 3% RH) wood blocks and stakes after treatment were labeled with durable tags. Treated non-durable wood species blocks and untreated blocks of heartwood of the durable species were exposed in the field in a ground proximity test following the AWPA E26 standard which is a covered, protected test that limits the leaching of water soluble wood preservatives such as borates. This test is used as a termite testing standard; however, we decided for this study to take both termite and decay ratings. It is a harsh test as it promotes both termite and wood decay attack close to but not in contact with soil. Treated stakes of both non-durable wood species and untreated stakes of heartwood of the durable species were exposed in the field using ground contact field stake tests according to the AWPA E7 standard. Each treatment was replicated five times for both tests and specimens were placed at field sites in Saucier, Mississippi, USA. All blocks and stakes were rated visually using a 0–10 scale as described in the AWPA standards (Table 2) after every year of installation for five years.

### 2.4. Statistical Analysis

Obtained data were averaged in Microsoft Excel and standard error of mean were calculated. All figures were prepared using GraphPad prism 7.

## 3. Results

### 3.1. Field Stake Tests

#### 3.1.1. Decay Ratings

**Figure 1:**
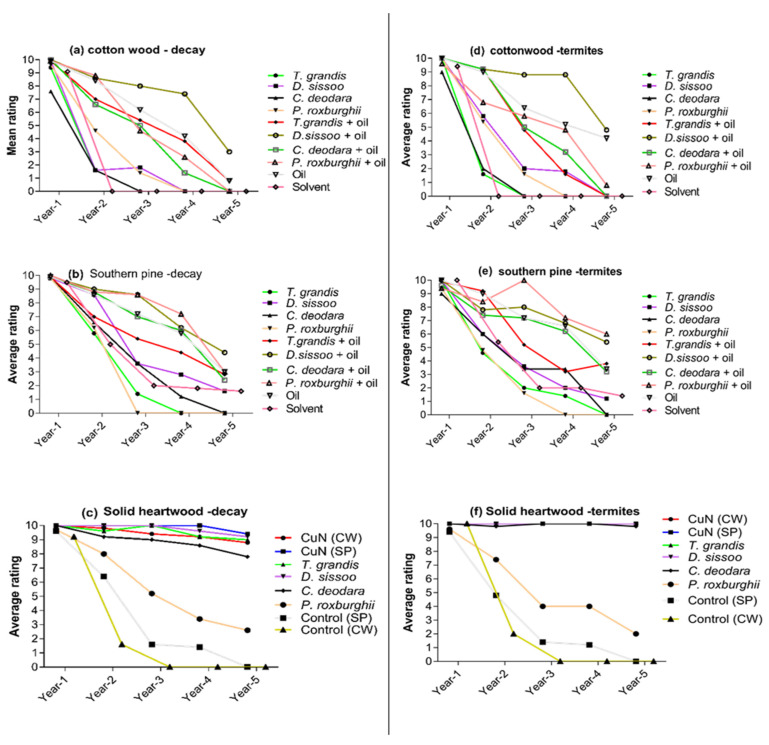
Average decay fungi damage rating for treated cottonwood (**a**), southern pine (**b**), untreated solid heartwood stakes (**c**), and average termite damage rating for treated cottonwood (**d**), southern pine (**e**), untreated solid heartwood stakes (**f**), exposed in the field for five years following the AWPA E7 ground contact test.

The average decay damage ratings for the cottonwood and southern pine test specimens in the ground contact field stake test (AWPA E7) exposure for five years are shown in Figure 1a,b and supplementary Table S1. Treated and untreated stakes of both non-durable wood species showed discoloration and softening associated with superficial microbial colonization after the first year of exposure. Ratings of all specimens differed non-significantly from each other with an average rating of 9 or above except cottonwood treated with *C. deodara* extract, which was moderately attacked by decay fungi (average rating 7.6). A considerable difference in damage by decay fungi in different treatments was observed after the second year of exposure. Solvent-treated cottonwood stakes failed within two years. Cottonwood stakes treated with *D. sissoo* extract or *P. roxburghii* extract failed after three years in the field. The majority of the treated cottonwood stakes were completely decayed after five years except stakes treated with *D. sissoo* extract + oil. The decay rate in southern pine stakes was somewhat less rapid than that observed for cottonwood. Untreated southern pine did not completely fail during the second year of study. Solvent-only treated stakes were severely attacked with an average rating of 5, similar to stakes treated with individual heartwood extract of *T. grandis*, *C. deodara*, and *P. roxburghii* with an average rating between 6 to 6.7. Southern pine stakes treated with linseed oil and heartwood extracts received slightly to moderate attack by decay fungi after two years.

After the third year of exposure, southern pine stakes treated with *P. roxburghii* extract and solvent had average ratings of 0–2, while stakes treated with a mixture of heartwood extract from all species and linseed oil in separate treatments received moderate to severe attack by decay fungi (6–8 average ratings). Southern pine stakes treated with extract of *T. grandis*, *C. deodara*, or *P. roxburghii* failed. Stakes treated with a mixture of oil and extracts were not completely decayed after five years of exposure, and overall, stakes treated with *D. sissoo* extract + oil received lower attack (>4 average ratings) by decay fungi than other oil mixture treatments (Figure 1a,b).

Solid heartwood stakes of four tested wood species and CuN treated cottonwood or southern pine were not attacked by decay fungi after the first year of exposure (Figure 1c). However, *T. grandis*, *D. sissoo*, and CuN treated stakes showed slight to superficial attack during the second to fifth-year exposure. In contrast, *C. deodara* stakes were moderately attacked after five years of exposure, while *P. roxburghii* stakes received moderate attack after the second year of exposure and were severely attacked after five years (Figure 1c; supplementary Table S3).

#### 3.1.2. Termite Ratings

The average termite damage ratings for the cottonwood and southern pine test specimens in the field stake test (AWPA E7) exposure for five years are shown in Figure 1d,e and supplementary Table S2. Termites slightly attacked cottonwood and southern pine stakes treated with *C. deodara* extract and *P. roxburghii* extract + oil after the first year of exposure. Termites did not attack solvent-treated southern pine stakes after a year of exposure, unlike solvent-treated cottonwood stakes that received slight attack. After the second year, termites severely attacked cottonwood stakes treated with *T. grandis* extract or *C. deodara* extract (<2 average ratings), while solvent-treated stakes failed (average rating 0). Stakes treated with linseed oil only, *T. grandis* extract + oil, *D. sissoo* extract + oil, and *C. deodara* extract + oil were slightly attacked after the second year of exposure. However, southern pine stakes treated with extracts of single species extracts of four test species were severely attacked by termites (5–6 average ratings) after the second year. Termites slightly attacked southern pine stakes treated with *T. grandis* + oil and oil only. Stakes of southern pine treated with *D. sissoo* extract + oil, *C. deodara* extract + oil or *P. roxburghii* + oil were moderately attacked after the second year of exposure. After five years in the field, cottonwood stakes treated with all treatment solutions were completely attacked by the termites (0–1 average ratings) except stakes treated with oil (average rating 4) and *D. sissoo* extract + oil (average ratings 5) which the termites attacked severely. Southern pine stakes treated with a mixture of oil and wood extracts of test species in separate treatment were moderately attacked after the third year and severely attacked after the fourth and fifth year of exposure to termites. Overall, stakes treated with heartwood extract of each species in separate treatments were more damaged by termites than the stakes treated with a mixture of oil + extract (Figure 1d,e).

**Figure 2:**
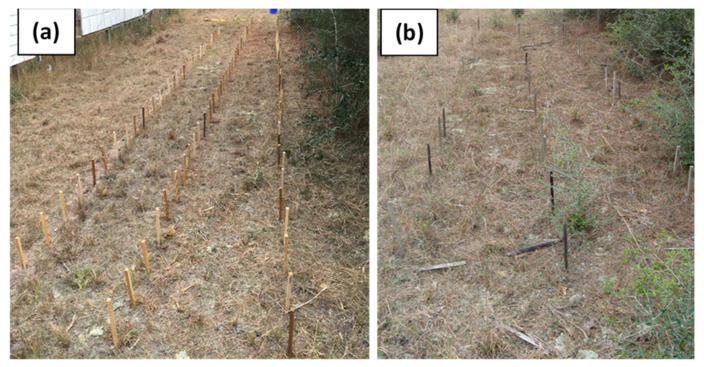
Example of array in AWPA E7 field stake ground contact test after installation in November 2015 (**a**) and after five years of exposure, showing treated and untreated samples in poor conditions (**b**).

Similar to decay fungi, solid heartwood stakes of durable species and CuN-treated cottonwood and southern pine stakes were not attacked by termites after the first year of exposure except *P. roxburghii* stakes which were slightly attacked by the termites (Figure 1f). However, this species′ stakes were moderately attacked after the second year of exposure and severely attacked by termites after the fourth. Termites destroyed stakes of *P. roxburghii* (average ratings 0) after five-year exposure while CuN treated cottonwood and southern pine stakes and stakes of other test species were sound after five years of exposure except for *C. deodara*, which received slight attack (rating > 9) at the end of the test period (Figure 1f; supplementary Table S3). The general condition of treated and untreated samples at the time of exposure and after five years is shown in Figure 2a,b.

### 3.2. Ground Proximity Test

#### 3.2.1. Decay Ratings

**Figure 3:**
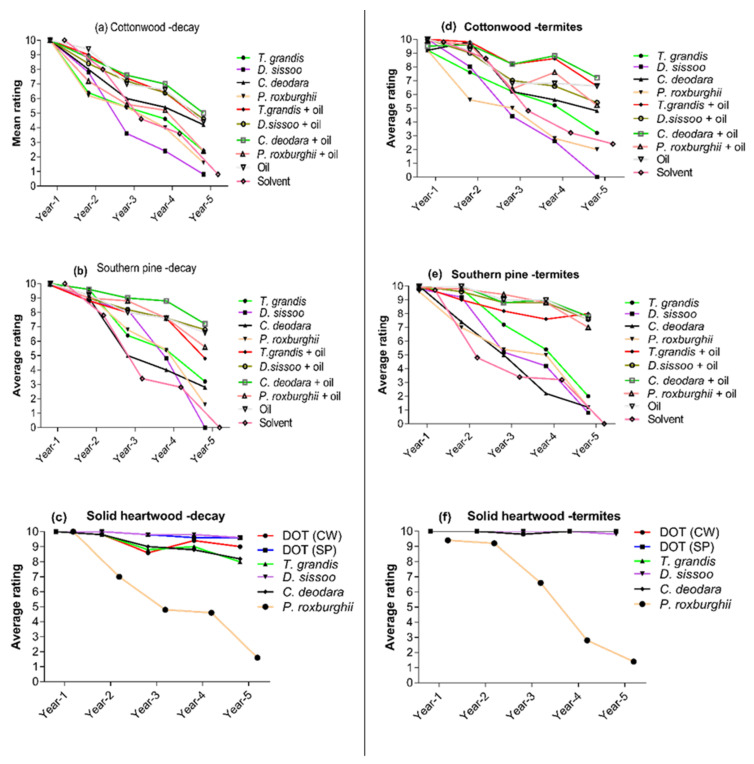
Average decay fungi damage rating for treated cottonwood (**a**), southern pine (**b**), untreated solid heartwood blocks (**c**), and average termite damage rating for treated cottonwood (**d**), southern pine (**e**), untreated solid heartwood blocks (**f**), exposed in the field for five years following the AWPA E26 ground proximity test.

Results of the ground proximity test showed that cottonwood and southern pine blocks were sound after one year of exposure (Figure 3a,b and supplementary Table S4). Cottonwood blocks treated with *T. grandis* extract and *T. grandis* extract + oil were slightly attacked after the second year of exposure. In comparison, cottonwood blocks treated with extracts of other species or extract + oil mixture in separate treatments were moderately attacked by decay fungi (average rating 6–8). After the second year of exposure, southern pine blocks showed moderate to slight attack (average ratings 8–9), except for solvent-treated blocks, which were moderately attacked by decay fungi. Cottonwood blocks treated with a mixture of extracts and oil or oil only showed moderate attack (average rating 7) after the third year of exposure except blocks treated with *P. roxburghii* extracts + oil. Cottonwood blocks treated with solvents or extracts only showed severe to very severe attack after the third, fourth, and fifth years of exposure in the field. Overall, blocks treated with *T. grandis* extract + oil, *D. sissoo* extract + oil, *C. deodara* extract + oil, or *C. deodara* extract showed lower damage than other treatments. Southern pine blocks treated with oil + wood extract of individual species showed moderate attack after second, third, and fourth years of exposure. In comparison, blocks treated with solvent or extract of individual species showed more damage during this period. After the fifth year of exposure, cottonwood blocks treated with *T. grandis* extract + oil, *D. sissoo* extract +oil, *C. deodara* extract, and *C. deodara* extract + oil were severely attacked by decay fungi (average rating < 5) while blocks in all other treatments were failed (average rating < 3) due to decay. Like cottonwood blocks, southern pine blocks treated with a mixture of oil and extract of individual heartwood extract showed lower damage than blocks treated with individual heartwood extract only after five years of exposure. Solvent-treated and *D. sissoo* extract-treated blocks of southern pine were completely decayed after five years of exposure (Figure 3a,b).

Decay fungi did not attack heartwood blocks of the four durable wood species or blocks of cottonwood or southern pine treated with boron (DOT) after the first year of exposure. However, boron-treated cottonwood blocks showed slight attack by decay fungi after the second, third, fourth, and fifth year of exposure (rating 9). Blocks of *T. grandis* and *D. sissoo* showed slight attack after the third, fourth, and fifth year of exposure. While decay fungi moderately attacked *C. deodara* blocks after five years of exposure and *P. roxburghii* blocks were severely attacked after this period (average rating 2) (Figure 3c; supplementary Table S6).

#### 3.2.2. Termite Ratings

Average termite damage ratings for cottonwood and southern pine blocks treated with heartwood extracts, linseed oil, or their mixture, exposed in the field using ground proximity tests, are presented in Figure 3d,e and supplementary Table S5. Cottonwood treated with individual extract of *T. grandis*, *C. deodara*, *P. roxburghii*, and *C. deodara* extract + oil showed a slight attack after one year of exposure. However, southern pine treated with *P. roxburghii* extract only showed a slight attack after one year of exposure. Cottonwood treated with a mixture of oil and individual heartwood extracts, or oil only performed better against termite attack throughout the exposure period. After five years of exposure, cottonwood blocks treated with *C. deodara* extract + oil or *T. grandis* extract + oil were moderately attacked with 10–30% of the cross-sectional area affected (average rating 7) with only 1 and 2 stakes which were severely attacked by the termite (average rating ≤ 6), respectively.

In comparison, blocks treated with solvent only or individual extract of *T. grandis*, *C. deodara*, or *P. roxburghii* in separate treatments were severely attacked (average rating < 5) by the termites after five years of exposure in the field, while blocks treated with *D. sissoo* extract were destroyed completely by termites. Like cottonwood, southern pine blocks treated with the mixture of oil and individual heartwood extracts of four species were less attacked by the termites than blocks treated with individual extracts only or solvents. After five years of exposure, southern pine blocks treated with a mixture of individual extract and oil only were moderately attacked by the termites with average ratings of 7 or above. Southern pine blocks treated with individual extracts of four species were severely attacked after five years of exposure with an average rating of 2 or less than 2, while the termites destroyed solvent-treated blocks after five years.

**Figure 4:**
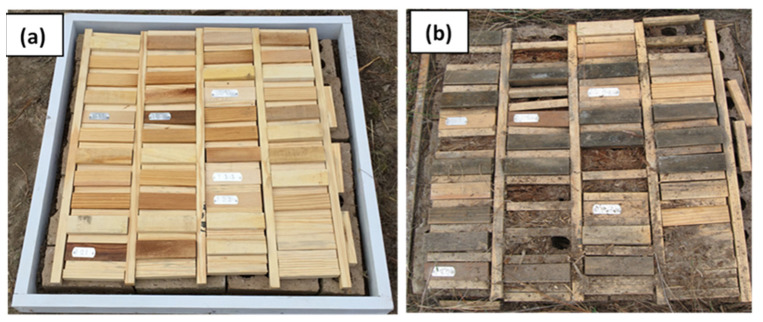
Example of an array in the AWPA E26 ground proximity test after installation in November 2015 (**a**) and after five years exposure, showing treated and untreated samples in poor conditions (**b**) (cover of test assembly not shown).

Heartwood blocks of durable wood species and boron (DOT) treated southern pine or cottonwood were sound after five years of exposure except for *P. roxburghii* blocks which showed slight attack after 1–2 year of exposure. Blocks of *P. roxburghii* were moderately attacked by the termite after three years and severely attacked after 4–5 years of exposure (Figure 3f). General condition of treated and un-treated samples at the time of exposure and after five years is shown the Figure 4a,b.

## 4. Discussion

Interest in the exploration and use of natural products as wood protectants is rapidly increasing around the world. Potential use of natural products as wood protectants against termites and decay fungi has been tested in many studies, however, most of the results in these studies are based on laboratory trials [24]. While laboratory trials are instrumental in testing the efficacy of wood preservatives, due to certain limitations, these must be supported by field testing to evaluate how test materials perform under natural conditions [25]. In the current study, we tested the combined preservative potential of heartwood extracts and linseed oil against decay fungi and termites in two field tests. Specifically, the sapwood of cottonwood and southern pine treated with heartwood extract, linseed oil, and an extract + oil mixture were exposed in ground proximity and ground contact field tests over five years. All samples were exposed at Harrison Experimental Forest, north of Gulfport, Mississippi (30°38′ N, 89°03′ W), which is within the American Wood Protection Association Deterioration Zone 5 and is considered a severe biodeterioration hazard zone. This site is dominated by pine forests and experiences a humid, subtropical climate. Predominate termites at the test location are *Reticulitermes* species, with *Reticulitermes flavipes* (Kollar) being the most common [26,27,28,29]. Compared to other economically important termite species (e.g., *Coptotermes* spp.), *Reticulitermes* spp. construct heavily branched and narrow underground tunnels that directly radiate from a central nest. They can travel up to 79 m in the branched and dense tunneling system, making them efficient locators of wood materials in a field setting [25]. Moreover, *R. flavipes* is resilient to temperature and soil conditions and can continue foraging to locate food resources during extreme environmental conditions [30]. These factors make them very efficient in finding wood samples in the field.

Results showed that cottonwood stakes from all treatment groups failed due to attack by decay fungi in ground contact exposure (AWPA E7). Comparatively higher ratings (less attack) were observed in stakes treated with *D. sissoo* extract + oil. While southern pine ground contact stakes treated with an extract + oil mixture of each species in separate treatments showed an average rating of 3–5 after five years of exposure, cottonwood stakes treated with *D. sissoo* extract + oil, or oil only, showed higher resistance against termites than decay fungi. Similarly, southern pine ground contact stakes treated with *P. roxburghii* extract + oil or *D. sissoo* extract + oil showed higher resistance against termites (≥6 average ratings) after five years of exposure. Heartwood stakes of *P. roxburghii* were not resistant (completely failed) to termites and decay after five years in ground contact. In the AWPA E26 ground proximity test, cottonwood blocks treated with all treatments (extract, oil, extract + oil) were severely attacked or failed due to decay fungi after five years. Comparatively higher resistance against decay fungi was observed in blocks treated with *D. sissoo* extract, *D. sissoo* extract + oil, *T. grandis* extract + oil or oil only. While southern pine blocks treated *C. deodara* extract + oil, or *D. sissoo* extract + oil showed higher resistance (moderately attacked) against decay fungi than other treatments. Overall, heartwood extract + oil better protected southern pine compared to cottonwood blocks after five years of exposure. Cottonwood blocks treated with *C. deodara* extract + oil provided increased resistance against termites compared to other treatments, but were severely attacked. While pinewood blocks treated with a mixture of extract and oil (in separate treatments) provided higher resistance against termites after five years of exposure. These treatments better protected southern pine than cottonwood. As with the AWPA E7 ground contact stakes test, *P. roxburghii* heartwood blocks failed due to termite and decay fungi attack.

Although samples were exposed to severe a deterioration hazard [28], stakes and blocks treated with CuN and DOT, respectively, performed well in this harsh environment. Stakes of both non-durable wood species treated with CuN were not attacked by termites after five years in the field. However, these showed slight to superficial attack by the decay fungi. While borate treated blocks in the ground proximity test showed very slight superficial signs of decay (decay rating > 9), they were not attacked by the termites after five years of exposure. It should be noted that even though the ground proximity (AWPA E26) method used in this study provides protection from precipitation (preventing borate leaching), it is an extremely harsh test for testing wood preservative and designed specifically for termite attack. We added decay ratings to the test as a modification of the AWPA E26 standard.

In previous studies, compounds present in the heartwood extracts of the four species tested showed strong biological activities against insects and fungi [31]. Chemical analyses of these extracts showed high concentrations of anthraquinone and squalene in *T. grandis*; three sesquiterpenes, cuprenene, himachalene, and cedrene in extracts of *C. deodara*; trimethoxyresveratrol in *D. sissoo*; and benzopyran from *P. roxburghii* heartwood [7,31]. Anthracenedione (anthraquinone) and squalene have been reported to have biocidal activity against termites and decay fungi. Similarly, sesquiterpenes are antifeedant, repellant, and illicit behavioral responses in subterranean termites. Our previous laboratory tests showed that non-durable wood species treated with heartwood extracts of these species were protected against *Reticulitermes flavipes* and *Heterotermes indicola* (Wasmann) at 10 mg mL^−1^ concentration [10,32,33]. These extracts are repellant, antifeedant, free radical scavengers, and found to be toxic to the symbiotic protozoa of these two termite species in laboratory tests [7,32]. Previous studies also showed that *T. grandis* extracts were effective against decay fungi [34]. Antifungal properties derived from extracts of *C. deodara* have been reported to be effective against *Trametes versicolor* (L.) Lloyd, *Aspergillus fumigatus* Fresen, and *Candida albicans* Berkh [33,35]. Similarly, extracts from *D. sissoo* showed antifungal activities against *Alternaria* and *Fusarium* species [36].

Previous studies showed that the addition of other chemicals might act synergistically with heartwood extracts to further increase toxicity against termites and decay fungi [5,10]. In the current study, we reduced extract concentration and combined them with linseed oil to exploit the synergy between extracts and oil in protecting non-durable wood species. We found that when linseed oil (20%) was mixed in extracts, it increased the extract′s efficacy as a wood protectant. Plant oils act as toxicants, repellents, and hydrophobic agents to protect the wood from decay fungi and termites [24]. Linseed oil is considered to have no toxic action against wood deteriorating organisms, but it has provided resistance to wood against termites and decay fungi in previous studies [14,18]. One possible mechanism is that the oil could be creating a hydrophobic barrier resulting in the displacement of water in woods with the oil treatment thus limiting biological attack [3,24]. Oil can transfer a toxicant further into wood for protection against termites and fungi [20]. In our previous studies, treatment of both non-durable wood species with *T. grandis* extract + oil or *D. sissoo* extract + oil prevented termite damage compared with the extract only treatments when AWPA E26 blocks and AWPA E7 stakes were exposed in the field for two years [10]. Linseed oil also ensures water repellency and dimensional stability of the treated wood, and in combination with boron, it protected wood against termites [37]. Several researchers have reported linseed oil or other oils as effective wood protecting agents in the laboratory and field studies. However, in the current study, it was not as effective as reported previously as wood treated with linseed oil only was severely attacked by the decay fungi and termites.

The current study showed that the combination of oil and heartwood extracts performed better in protecting wood than the extracts alone. However, these were as not effective as oil-based copper naphthenate (CuN) or water-based disodium octaborate tetrahydrate (DOT) in ground contact and protected ground proximity tests, respectively. This may be due to the tested extract or oil concentrations being below the required threshold for effectiveness against termites and decay fungi. Plant oils and extracts of durable heartwood species are used in crude or semi crude form, and formulation based on these products is not available commercially. Cost and stability of the extractives are the primary concerns. In an earlier study, we showed that non-durable wood treated with extracts of *C. deodara* + oil showed lower resistance to termites due to the leaching of chemicals from wood [10].

## 5. Conclusions

Results of the current study indicated that extracts combined with linseed oil seemed to add some protection from termite and decay attack to the treated non-durable wood species in field exposure tests compared to untreated or extract only treated non-durable wood. However, these were not as effective as synthetic preservatives (CuN or DOT) in protecting non-durable wood species. Future studies should examine single-extract component isolates combined with oils that will ultimately lead to new chemistries for industrialized wood preservative development.

## Figures and Tables

**Table 1:** Retention of treatment solutions in southern pine and cottonwood.

Treatment Solution	Retentions (Kg/m^3^)—AWPA E7 Stakes		Retentions (Kg/m^3^)—AWPA E26 Blocks	
	Cotton Wood (Mean ± StDev)	Southern Pine (Mean ± StDev)	Cotton Wood (Mean ± StDev)	Southern Pine (Mean ± StDev)
*T. grandis*	557.59 ± 25.34	501.27 ± 61.09	408.02 ± 47.09	351.09 ± 14.48
*D. sissoo*	564.69 ± 24.69	479.83 ± 93.69	471.78 ± 62.25	362.46 ± 10.14
*C. deodara*	580.72 ± 33.16	506.29 ± 41.47	469.93 ± 56.62	363.71 ± 38.58
*P. roxburghii*	571.49 ± 52.87	529.07 ± 23.54	453.51 ± 60.72	362.01 ± 29.79
*T. grandis* + oil	580.48 ± 36.56	498.42 ± 27.21	487.57 ± 89.62	388.64 ± 28.41
*D. sissoo* + oil	579.27 ± 58.61	517.18 ± 38.06	506.29 ± 60.66	389.19 ± 45.86
*C. deodara* + oil	536.56 ± 31.38	484.46 ± 51.81	524.03 ± 44.20	403.73 ± 40.78
*P. roxburghii* + oil	546.95 ± 28.04	518.16 ± 25.36	372.98 ± 38.18	456.12 ± 35.38
Oil (linseed)	570.03 ± 24.31	489.52 ± 59.81	458.96 ± 84.49	394.86 ± 39.52
CuN (in oil)	398.7 ± 32.0	447.5 ± 22.9	N/A	N/A
Borate (DOT)	N/A	N/A	865.73 ± 55.20	623.40 ± 21.60
Solvent	585.69 ± 25.31	512.79 ± 33.78	487.68 ± 35.47	375.47 ± 69.91

**Table 2:** AWPA E7 and E26 visual termite and decay damage rating scheme.

10	Sound, no decay or insect damage
9.5	Trace, surface nibbles permitted
9	Slight attack, up to 3% of cross-sectional area affected
8	Moderate attack, 3–10% of cross-sectional area affected
7	Moderate/severe attack, penetration, 10–30% of cross-sectional area affected
6	Severe attack, 30–50% of cross-sectional area affected
4	Very severe attack, 50–75% of cross-sectional area affected
0	Failure

## Data Availability

All data are available in the article.

## Supplementary Materials

The following are available online at https://www.mdpi.com/article/10.3390/insects12090803/s1. Table S1: Average decay fungi damage rating for treated cottonwood and southern pine exposed in the field for five years following the AWPA E7 ground contact test. Table S2. Average termite damage rating for treated cottonwood and southern pine exposed in the field for five years following the AWPA E7 ground contact test. Table S3. Average decay fungi and termite damage rating for untreated solid heartwood stakes exposed in the field for five years following the AWPA E7 ground contact test. Table S4. Average decay fungi damage rating for treated cottonwood and southern pine exposed in the field for five years following the AWPA E26 ground proximity test. Table S5. Average termite damage rating for treated cottonwood and southern pine exposed in the field for five years following the AWPA E26 ground proximity test. Table S6. Average decay fungi and termite damage rating for untreated solid heartwood blocks exposed in the field for five years following the AWPA E 26 ground proximity test.
