# Supplementary material for: Evaluation of Heartwood Extracts Combined with Linseed Oil as Wood Preservatives in Field Tests in Southern Mississippi, USA
Source: Insects. 2021 Sep 8;12(9):803. doi: 10.3390/insects12090803 (PMC8467827; doi:10.3390/insects12090803)
Supplement: Supplementary file 1 [file insects-12-00803-s001.zip › insects-1365394-supplementary.pdf]

Tables showing the average decay fungi and termite damage rating of wood stakes and blocks following exposure in the field using two tests

Table S1: Average decay fungi damage rating for treated cottonwood and southern pine exposed in the field for five years following the AWPA E7 ground contact test.

Treatments	Southern pine					Cottonwood				
	Year 1	Year 2	Year 3	Year 4	Year 5	Year 1	Year 2	Year 3	Year 4	Year 5
T. grandis	9.9 (0.1)	5.8 (1.7)	1.4 (1.4)	0 (0)	0 (0)	9.9 (0.1)	1.6 (1.6)	0 (0)	0 (0)	0 (0)
D. sissoo	9.8 (0.1)	8.6 (0.2)	3.6 (2.2)	2.8 (1.7)	1.6 (1.6)	9.9 (0.1)	1.6 (1.6)	1.8 (1.8)	0 (0)	0 (0)
C. deodara	9.9(0.1)	6.6 (1.6)	3.6 (2.2)	1.2 (1.2)	0 (0)	7.6 (1.9)	1.6 (1.6)	0 (0)	0 (0)	0 (0)
P. roxburghii	9.8 (0.1)	6.2 (1.6)	0 (0)	0 (0)	0 (0)	9.6 (0.1)	4.6 (1.9)	1.4 (1.4)	0 (0)	0 (0)
T. grandis + oil	9.9 (0.1)	7.0 (1.8)	5.4 (2.2)	4.4 (1.8)	2.8 (1.7)	9.9 (0.1)	7 (1.7)	5.4 (1.3)	3.8 (1.5)	0.8 (0.8)
D. sissoo + oil	9.9 (0.1)	9 (0)	8.6 (0.1)	6.2 (1.5)	4.4 (1.7)	10 (0)	8.6 (0.2)	8 (0.3)	7.4 (0.5)	3 (1.3)
C. deodara + oil	9.9(0.1)	8.8 (0.2)	7 (1.7)	6 (1.5)	2.4 (1.6)	10 (0)	6.6 (1.7)	5 (2.0)	1.4 (1.4)	0 (0)
P. roxburghii + oil	10 (0)	8.8 (0.2)	8.6 (0.2)	7.2 (0.5)	3 (1.9)	9.9 (0.1)	8.8 (0.2)	4.6 (1.2)	2.6 (1.6)	0 (0)
Oil	9.9 (0.1)	8.6 (0.5)	7.2 (1.8)	5.8 (1.53)	3 (1.3)	9.9 (0.1)	8.4 (0.2)	6.2 (1.6)	4.2 (1.7)	0.8 (0.8)
Solvent	9.5 (0.1)	5 (2.0)	2 (2.0)	1.8 (1.8)	1.6 (1.6)	9.1 (0.3)	0 (0)	0 (0)	0 (0)	0 (0)

Values represent averages of five stakes rating and values in parenthesis represents standard error of mean

Table S2. Average termite damage rating for treated cottonwood and southern pine exposed in the field for five years following the AWPA E7 ground contact test.

Treatments	Southern pine					Cottonwood				
	Year 1	Year 2	Year 3	Year 4	Year 5	Year 1	Year 2	Year 3	Year 4	Year 5
T. grandis	10 (0.0)	4.6 (1.9)	2 (2.0)	1.4 (1.4)	0 (0)	10 (0)	1.6 (1.6)	0 (0)	0 (0)	0 (0)
D. sissoo	10 (0.0)	6 (2.4)	3.6 (2.2)	2 (2.0)	1.2 (1.2)	10 (0)	5.8 (2.3)	2 (2.0)	1.8 (1.8)	0 (0)
C. deodara	9.0 (0.7)	6 (2.4)	3.4 (2.1)	3.4 (2.0)	0 (0)	9 (0.6)	2 (2.0)	0 (0)	0 (0)	0 (0)
P. roxburghii	9.4 (0.6)	4.8 (2.0)	1.6 (1.6)	0 (0)	0 (0)	10 (0)	5.4 (2.2)	1.6 (1.6)	0 (0)	0 (0)
T. grandis + oil	9.9 (0.1)	8.4 (0.6)	5.2 (2.1)	3.2 (1.9)	3.8 (2.3)	10 (0)	9.2 (0.3)	4.8 (2.0)	1.6 (1.6)	0 (0)
D. sissoo + oil	10 (0.0)	7.8 (0.5)	8 (2.0)	6.8 (1.7)	5.4 (2.2)	10 (0)	9.2 (0.5)	8.8 (0.3)	8.8 (0.3)	4.8 (1.6)
C. deodara + oil	9.6 (0.4)	7.4 (1.9)	7.2 (1.8)	6.2 (1.5)	3.2 (1.3)	10 (0)	9.2 (0.8)	5 (2.1)	3.2 (2.0)	0 (0)
P. roxburghii + oil	9.4 (0.6)	8.4 (2.2)	10 (0)	7.2 (1.8)	6 (2.4)	9.6 (0.4)	6.8 (1.8)	5.8 (2.3)	4.8 (1.9)	0.8 (0.8)
Oil	10 (0.0)	9 (0.6)	7.2 (1.8)	6.6 (1.6)	3. 4 (2.0)	10 (0)	9 (0.6)	6.4 (1.6)	5.2 (2.1)	4.2 (1.9)
Solvent	10 (0.0)	5.4 (2.2)	2 (2.0)	2 (2.0)	1.4 (1.4)	9.4 (0.6)	0 (0)	0 (0)	0 (0)	0 (0)

Values represent averages of five stakes rating and values in parenthesis represents standard error of mean

Table S3. Average decay fungi and termite damage rating for untreated solid heartwood stakes exposed in the field for five years following the AWPA E7 ground contact test.

Treatments	Decay fungi					Termites				
	Year 1	Year 2	Year 3	Year 4	Year 5	Year 1	Year 2	Year 3	Year 4	Year 5
CuN (CW)	10 (0)	9.8 (0.2)	9.4 (0.2)	9.2 (0.2)	8.8 (0.3)	10 (0)	10 (0)	10 (0)	10 (0)	10 (0)
CuN (SP)	10 (0)	10 (0)	10 (0)	10 (0)	9.4 (0.2)	10 (0)	10 (0)	10 (0)	10 (0)	10 (0)
T. grandis	10 (0)	9.6 (0.2)	10 (0)	9.2 (0.2)	9 (0)	10 (0)	10 (0)	10 (0)	10 (0)	10 (0)
D. sissoo	10 (0)	10 (0)	10 (0)	9.6 (0.2)	9.2 (0.2)	10 (0)	10 (0)	10 (0)	10 (0)	10 (0)
C. deodara	10 (0)	9.2 (0.2)	9 (0.3)	8.6 (0.4)	7.8 (0.3)	10 (0)	9.8 (0.2)	10 (0)	10 (0)	9.8 (0.2)
P. roxburghii	9.7 (0.1)	8 (0.3)	5.2 (2.1)	3.4 (2.0)	2.6 (1.6)	9.6 (0.4)	7.4 (1.9)	4 (2.4)	4 (2.4)	2 (2.0)
Control (SP)	9.6 (0.1)	6.4 (1.6)	1.6 (1.6)	1.4 (1.4)	0 (0)	9.4 (0.6)	4.8 (2.2)	1.4 (1.4)	1.2 (1.2)	0 (0)
Control (CW)	9.2 (0.3)	1.6 (1.6)	0 (0)	0 (0)	0 (0)	10 (0)	2 (2.0)	0 (0)	0 (0)	0 (0)

Values represent averages of five stakes rating and values in parenthesis represents standard error of mean; CW: cottonwood; SP: southern pine; CuN: copper naphthenate

Table S4. Average decay fungi damage rating for treated cottonwood and southern pine exposed in the field for five years following the AWPA E26 ground proximity test.

Treatments	Southern pine					Cottonwood				
	Year 1	Year 2	Year 3	Year 4	Year 5	Year 1	Year 2	Year 3	Year 4	Year 5
T. grandis	10 (0)	9.6 (0.4)	6.4 (1.7)	5.4 (1.6)	3.2 (1.9)	10 (0)	6.4 (1.6)	5.4 (1.3)	4.6 (1.2)	2.4 (0.9)
D. sissoo	10 (0)	8.8 (0.2)	8.2 (0.3)	4.8 (1.9)	0 (0)	10 (0)	7.8 (0.2)	3.6 (1.5)	2.4 (0.9)	0.8 (0.8)
C. deodara	10 (0)	9 (0.6)	5 (2.0)	4 (1.8)	2.8 (1.7)	10 (0)	8 (1.0)	6 (1.5)	5.4 (1.5)	4.2 (1.2)
P. roxburghii	10 (0)	9.2 (0.3)	6.8 (1.7)	5.4 (1.4)	1.6 (0.9)	10 (0)	6.2 (1.6)	5.4 (1.3)	4 (1.1)	1.6 (0.9)
T. grandis + oil	9.9 (0.1)	8.8 (0.3)	8 (0.3)	7.6 (0.6)	4.8 (1.3)	10 (0)	9 (0.5)	7.4 (0.8)	6.4 (1.6)	4.4 (1.3)
D. sissoo + oil	10 (0)	9 (0.3)	8.2 (0.5)	7.6 (0.4)	6.8 (0.7)	10 (0)	8.4 (0.4)	7.2 (0.4)	6.4 (0.7)	4.6 (0.6)
C. deodara + oil	10 (0)	9.6 (0.2)	9 (0)	8.8 (0.2)	7.2 (0.9)	10 (0)	8 (0.2)	7.6 (0.9)	7 (0.4)	5 (0.6)
P. roxburghii + oil	10 (0)	9 (0.4)	8.8 (0.8)	7.6 (0.9)	5.6 (0.6)	10 (0)	7.2 (0.8)	5.6 (0.6)	5.2 (0.7)	2.4 (0.9)
Oil	10 (0)	9.2 (0.4)	8 (0.6)	7.6 (1.0)	6.6 (0.8)	10 (0)	9.4 (0.2)	7 (0.3)	6.6 (0.2)	4.4 (1.1)
Solvent	10 (0)	7.8 (0.5)	3.4 (2.0)	2.8 (1.7)	0 (0)	10 (0)	8 (0.4)	4.6 (1.2)	3.6 (0.9)	0.8 (0.8)

Values represent averages of five blocks rating and values in parenthesis represents standard error of mean

Table S5. Average termite damage rating for treated cottonwood and southern pine exposed in the field for five years following the AWPA E26 ground proximity test.

Treatments	Southern pine					Cottonwood				
	Year 1	Year 2	Year 3	Year 4	Year 5	Year 1	Year 2	Year 3	Year 4	Year 5
T. grandis	9.8 (0.2)	9.8 (0.2)	7.2 (0.7)	5.4 (2.2)	2 (2.0)	9.2 (0.8)	7.6 (1.9)	6.2 (1.6)	5.2 (1.3)	3.2 (1.3)
D. sissoo	9.8 (0.2)	9.2 (0.5)	5.2 (2.1)	4.2 (1.8)	08 (0.8)	10 (0)	8 (2.0)	4.4 (1.8)	2.6 (1.7)	0 (0)
C. deodara	10 (0)	7.4 (1.9)	5 (2.1)	2.2 (1.4)	1.2 (1.2)	9.2 (0.8)	9.8 (0.2)	6.2 (1.5)	5.6 (1.6)	4.8 (1.9)
P. roxburghii	9.6 (0.2)	7 (1.8)	5.4 (1.5)	5 (1.4)	1.2 (1.2)	9.2 (0.5)	5.6 (1.4)	5 (1.3)	2.8 (1.7)	2 (1.2)
T. grandis + oil	10 (0)	9 (0.4)	8.2 (0.3)	7.6 (1.0)	8 (0.3)	10 (0)	9.8 (0.2)	8.2 (0.5)	8.6 (0.6)	6.6 (1.7)
D. sissoo + oil	10 (0)	9.6 (0.4)	8.8 (0.5)	8.8 (0.5)	7.6 (0.8)	10 (0)	9 (0.6)	7 (0.4)	6.6 (0.6)	5.4 (0.8)
C. deodara + oil	10 (0)	10 (0)	8.8 (0.3)	9 (0.4)	7.8 (0.6)	9.5 (0.2)	9.6 (0.2)	8.2 (0.3)	8.8 (0.3)	7.2 (0.3)
P. roxburghii + oil	9.9 (0.1)	9.8 (0.2)	9.4 (0.4)	8.8 (0.3)	7 (0.3)	9.9 (0.1)	9.6 (0.4)	6.4 (0.6)	7.6 (0.6)	5.2 (1.3)
Oil	10 (0)	10 (0)	9 (0.3)	9 (0.4)	7.6 (0.4)	10 (0)	9.2 (0.8)	6.8 (0.9)	6.8 (0.3)	6.6 (0.7)
Solvent	9.7 (0.2)	4.8 (2.2)	3.4 (2.1)	3.2 (2.0)	0 (0)	9.8 (0.2)	8.6 (0.6)	4.8 (1.3)	3.2 (1.3)	2.4 (1.6)

Values represent averages of five blocks rating and values in parenthesis represents standard error of mean

Table S6. Average decay fungi and termite damage rating for untreated solid heartwood blocks exposed in the field for five years following the AWPA E 26 ground proximity test.

**Treatments**	**Decay fungi**					**Termites**				
	Year 1	Year 2	Year 3	Year 4	Year 5	Year 1	Year 2	Year 3	Year 4	Year 5
DOT (CW)	10 (0)	9.8 (0.2)	8.6 (0.2)	9.4 (0.4)	9 (0.3)	10 (0)	10 (0)	9.8 (0.2)	10 (0)	10 (0)
DOT (SP)	10 (0)	10 (0)	9.8 (0.2)	9.6 (0.2)	9.6 (0.2)	10 (0)	10 (0)	10 (0)	10 (0)	10 (0)
T. grandis	10 (0)	9.8 (0.2)	8.8 (0.3)	9 (0.3)	8 (0.6)	10 (0)	10 (0)	10 (0)	10 (0)	10 (0)
D. sissoo	10 (0)	10 (0)	9.8 (0.2)	9.8 (0.2)	9.6 (0.4)	10 (0)	10 (0)	10 (0)	10 (0)	9.8 (0.2)
C. deodara	10 (0)	9.8 (0.2)	9 (0)	8.8 (0.2)	8.2 (0.2)	10 (0)	10 (0)	9.8 (0.2)	10 (0)	10 (0)
P. roxburghii	10 (0)	7 (1.9)	4.8 (1.9)	4.6 (1.8)	1.6 (1.6)	9.4 (0.4)	9.2 (0.3)	6.6 (1.7)	2.8 (1.9)	1.4 (1.4)

Values represent averages of five blocks rating and values in parenthesis represents standard error of mean; CW: cottonwood; SP: southern pine; DOT: disodium octaborate tetrahydrate
